# Supplementary material for: Structural characterization of encapsulated ferritin provides insight into iron storage in bacterial nanocompartments
Source: eLife. 2016 Aug 16;5:e18972. doi: 10.7554/eLife.18972 (PMC5012862; doi:10.7554/eLife.18972)
Supplement: Supplementary file 1. — The amino acid sequences of ferritin family proteins were aligned progressively using EMBL-EBI web services (Sutter et al., 2008; Akita et al., 2007) , Clustal Omega (McHugh et al., 2014) T-Coffee (Contreras et al., 2014) and MAFFT (Helgstrand et al., 2003). Protein names were adapted from either UniprotKB (Sutter et al., 2008), KEGG (Roberts et al., 2011) database or common name with PDB entry code (He and Marles-Wright, 2015). Sequences were sorted in an order corresponding to the clades in phylogenetic tree (Figure 13). The alignment was edited by Espript 3.0 web server (Aberg et al., 1993). The R. rubrum EncFtn (Rru_A0973) sequence ishighlighted in yellow. The ferroxidase centre (FOC) of Pseudo-nitzschia multiseries ferritin (PmFtn_4ITW) (highlighting in blue) consists of FeA site (E16, E49, E52) and FeB site (E49, E95, E131, Q128) which are labelled with solid red triangles (Grant et al., 1998). Another iron binding site in PmFtn_4ITW (the gateway site or FeC site [Bradley et al., 2014]) consists of E48, E45 and E131 which are marked with solid blue circles (Grant et al., 1998). The FOC of R. rubrum EncFtn is labelled with empty red triangles as E32, E62, H65 and Y39; and the iron entry site is marked with empty blue circles including E31 and E34. The putative iron exit site is marked with empty blue squares including H57, E61 and E64. The C-terminal localization sequences common to the encapsulin associated ferritins are highlighted within the red rectangle. DOI: http://dx.doi.org/10.7554/eLife.18972.035 [file elife-18972-supp1.pdf]

Supplementary file 1. Multiple sequence alignment

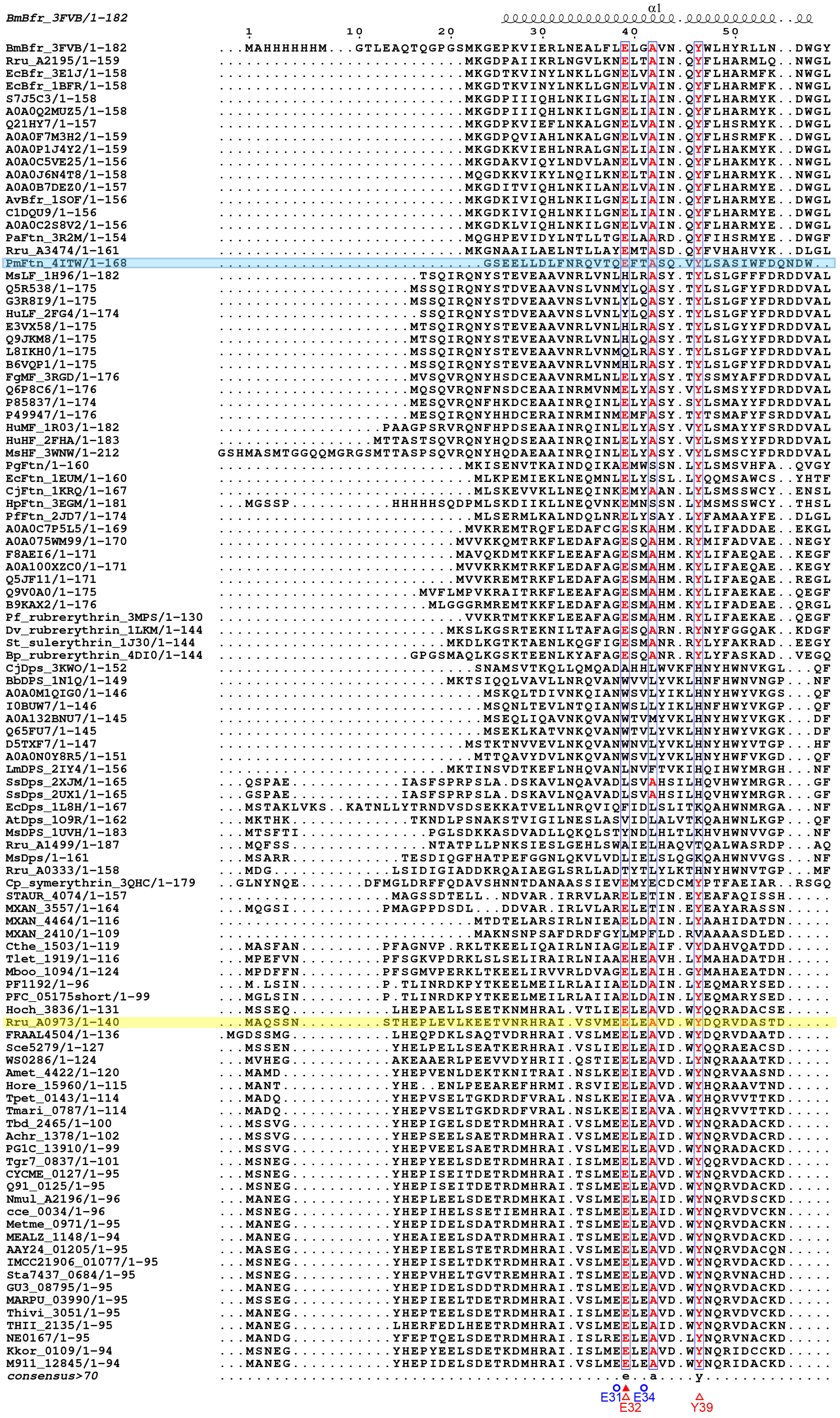
BmBfr_3FVB/1-182
α1
1
10
20
30
40
50
consensus>70
E31
E32
E34
Y39

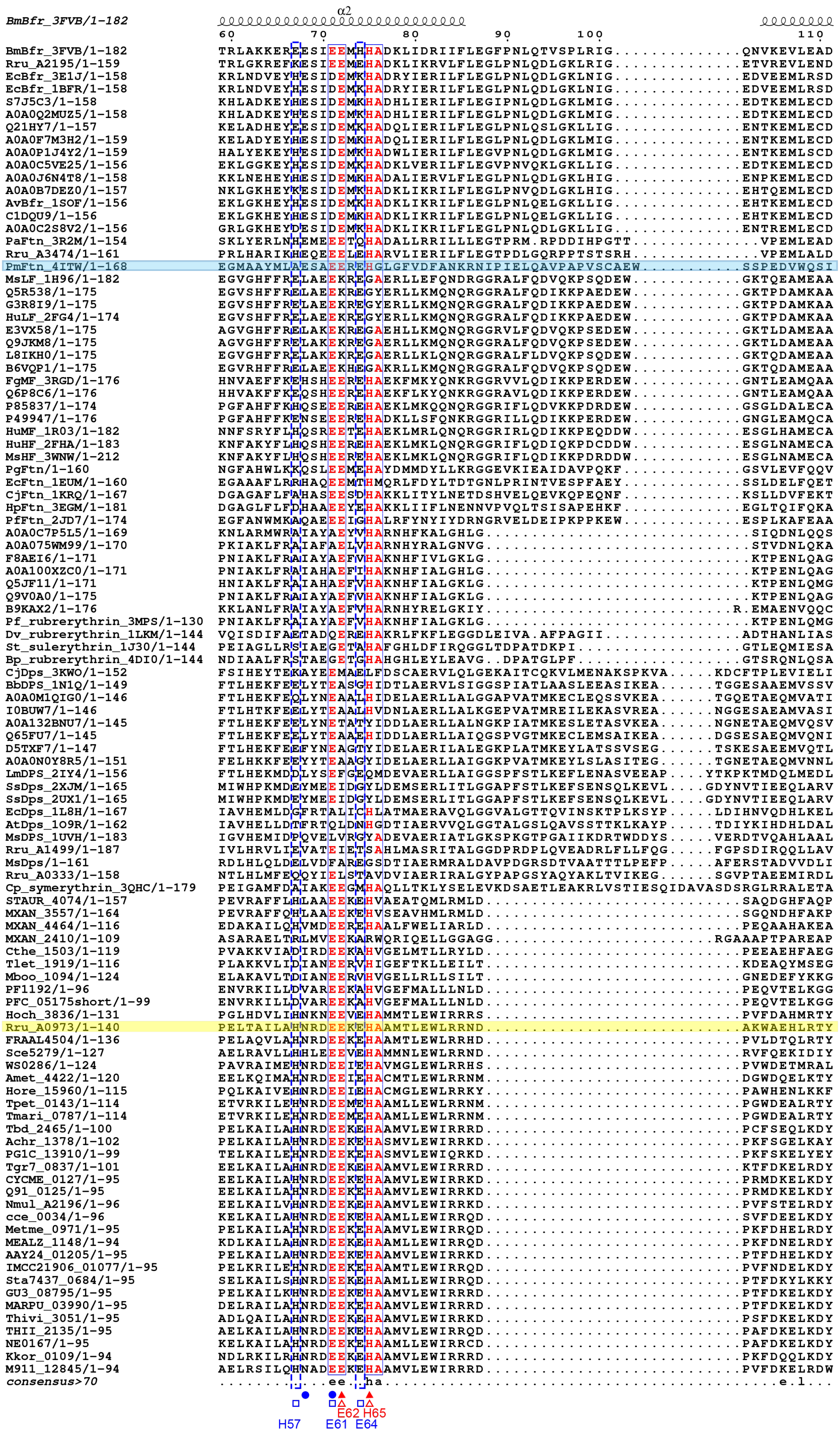
BmBfr_3FVB/1-182
α2
60 70 80 90 100 110
BmBfr_3FVB/1-182 TRLAKKEREESIEEMHHADKLIDRIIFLEGFPNLQTVSPLRIG..............QNVKEVLEAD
Rru_A2195/1-159 TRLGKREFKESIEEMEHADKLIKRVLFLEGLPNLQDLGKLRIG..............ETVREVLEND
EcBfr_3E1J/1-158 KRLNDVEYHESIDEMKHADRYIERILFLEGLPNLQDLGKLNIG..............EDVEEMLRSD
EcBfr_1BFR/1-158 KRLNDVEYHESIDEMKHADRYIERILFLEGLPNLQDLGKLNIG..............EDVEEMLRSD
S7J5C3/1-158 KHLADKEYHESIDEMKHADHLIERILFLEGIPNLQDLGKLMIG..............EDTKEMLECD
A0A0Q2MUZ5/1-158 KHLADKEYHESIDEMKHADHLIERILFLEGVPNLQDLGKLMIG..............EDTKEMLECD
Q21HY7/1-157 KELADHEYEESIDEMKHADQLIERILFLEGLPNLQSLGKLLIG..............ENTKEMLECD
A0A0F7M3H2/1-159 KELADYEYHESIDEMKHADQLIERILFLEGLPNLQDLGKLMIG..............ENTKEMLECD
A0A0P1J4Y2/1-159 HALYEKEYHESIDEMKHADWLIERILFLEGVPNLQDLGKLMIG..............ENTKEMLSCD
A0A0C5VE25/1-156 EKLGGKEYHESIDEMKHADKLVERILFLEGVPNVQKLGKLLIG..............EDTKEMLECD
A0A0J6N4T8/1-158 KKLNEHEYHESIDEMKHADALIERVLFLEGLPNLQDLGKLHIG..............ENPKEMLECD
A0A0B7DEZ0/1-157 NKLGKHEYKESIDEMKHADKLIKRILFLEGLPNVQDLGKLHIG..............EHTQEMLECD
AvBfr_1SOF/1-156 EKLGKHEYHESIDEMKHADKLIKRILFLEGLPNLQELGKLLIG..............EHTKEMLECD
C1DQU9/1-156 EKLGKHEYHESIDEMKHADKLIKRILFLEGLPNLQELGKLLIG..............EHTKEMLECD
A0A0C2S8V2/1-156 GRLGKHEYDESIDEMKHADKLIKRILFLEGLPNLQDLGKLLIG..............ENTREMLECD
PaFtn_3R2M/1-154 SKLYERLNHEMEEETQHADALLRRILLLEGTPRM.RPDDIHPGTT...............VPEMLEAD
Rru_A3474/1-161 PRLHARIKHEQEEELEHARVLIERILFLEGTPDLGQRPPTSTSRH...............VPEMLALD
PmFtn_4ITW/1-168 EGMAAYMLAESAEEREHGLGFVDFANKRNIPIELQAVPAPVSCAEW...........SSPEDVWQSI
MsLF_1H96/1-182 EGVGHFFRELAEEKREGAERLLEFQNDRGGRALFQDVQKPSQDEW...........GKTQEAMEAA
Q5R538/1-175 EGVSHFFRELAEEKREGYERLLKMQNQRGGRALFQDIKKPAEDEW...........GKTPDAMKAA
G3R8I9/1-175 EGVSHFFRELAEEKREGYERLLKMQNQRGGRALFQDIKKPAEDEW...........GKTPDAMKAA
HuLF_2FG4/1-174 EGVSHFFRELAEEKREGYERLLKMQNQRGGRALFQDIKKPAEDEW...........GKTPDAMKAA
E3VX58/1-175 AGVGHFFRELAKEKREGAEHLLKMQNQRGGRVLFQDVQKPSEDEW...........GKTLDAMEAA
Q9JKM8/1-175 AGVGHFFRELAKEKREGAERLLKMQNQRGGRALFQDVQKPSEDEW...........GKTLDAMEAA
L8IKH0/1-175 EGVGHFFRELAKEKREGAERLLKLQNQRGGRALFLDVQKPSEDEW...........GKTQDAMEAA
B6VQP1/1-175 EGVRHFFRELAEEKHEGAKRLLKMQNQRGGRALFQDVQKPSQDEW...........GKTQDAMEAA
FgMF_3RGD/1-176 HNVAEFFKEHSHEEREHAEKFMKYQNKRGGRVVLQDIKKPERDEW...........GNTLEAMQAA
Q6P8C6/1-176 HHVAKFFKEQSHEEREHAEKFLKYQNKRGGRAVLQDIKKPERDEW...........GNTLEAMQAA
P85837/1-174 PGFAHFFKHQSEEEREHAEKLMKQQNQRGGRIFLQDVKKPDRDEW...........GSGLDALECA
P49947/1-176 PGFAHFFKENSEEEREHADKLLSFQNKRGGRILLQDIKKPERDEW...........GNGLEAMQCA
HuMF_1R03/1-182 NNFSRYFLHQSREETEHAEKLMRLQNQRGGRIRLQDIKKPEQDDW...........ESGLHAMECA
HuHF_2FHA/1-183 KNFAKYFLHQSHEEREHAEKLMKLQNQRGGRIFLQDIQKPDCDDW...........ESGLNAMECA
MsHF_3WNW/1-212 KNFAKYFLHQSHEEREHAEKLMKLQNQRGGRIFLQDIKKPDRDDW...........ESGLNAMECA
PgFtn/1-160 NGFAHWLKKQSLEEMEHAYDMMDYLLKRGGEVKIEAIDAVPQKF............GSVLEVFQQV
EcFtn_1EUM/1-160 EGAAAFLRRHAQEEMTHMQRLFDYLTDTGNLPRINTVESPFAEY............SSLDELFQET
CjFtn_1KRQ/1-167 DGAGAFLFAHASEESDHAKKLITYLNETDSHVELQEVKQPEQNF............KSLLDVFEKT
HpFtn_3EGM/1-181 DGAGLFLFDHAAEEYEHAKKLIIFLNENNVPVQLTSISAPEHKF............EGLTQIFQKA
PfFtn_2JD7/1-174 EGFANWMKAQAEEEIGHALRFYNYIYDRNGRVELDEIPKPPKEW............ESPLKAFEAA
A0A0C7P5L5/1-169 KNLARMWRAIAYAEYVHARNHFKALGHLG............................SIQDNLQQS
A0A075WM99/1-170 PKIAKLFRAIAFAELVHARNHYRALGNVG............................STVDNLQKA
F8AEI6/1-171 PNIAKLFRAIAYAEFVHAKNHFIVLGKLG............................KTPENLQAG
A0A100XZC0/1-171 PNIAKLFRAIAHAEFIHAKNHFIALGHLG............................KTPENLQAG
Q5JF11/1-171 HNIAKLFRAIAHAEFVHAKNHFIALGKLG............................KTPENLQMG
Q9V0A0/1-175 PNIAKLFRAIAYAEFVHAKNHFIALGKLG............................KTPENLQAG
B9KAX2/1-176 KKLANLFRAIAYAEFVHARNHYRELGKIY...........................R.EMAENVQQC
Pf_rubrerythrin_3MPS/1-130 PNIAKLFRAIAYAEFVHAKNHFIALGKLG............................KTPENLQMG
Dv_rubrerythrin_1LKM/1-144 VQISDIFAETADQEREHAKRLFKFLEGGDLEIVA.AFPAGII..............ADTHANLIAS
St_sulerythrin_1J30/1-144 PEIAGLLRSIAEGETAHAFGHLDFIRQGGLTDPATDKPI..................GTLEQMIESA
Bp_rubrerythrin_4DI0/1-144 NDIAALFRSTAEGETGHAHGHLEYLEAVG..DPATGLPF..................GTSRQNLQSA
CjDps_3KWO/1-152 FSIHEYTEKAYEEMAELFDSCAERVLQLGEKAITCQKVLMENAKSPKVA.....KDCFTPLEVIELI
BbDPS_1N1Q/1-149 FTLHEKFEELYTEASGHIDTLAERVLSIGGSPIATLAASLEEASIKEA......TGGESAAEMVSSV
A0A0M1QIG0/1-146 FTLHEKFEQLYNEAALHIDELAERLLALGGAPVATMKECLEQSSVKEA......TGGQETAEQMVATI
I0BUW7/1-146 FTLHTKFEELYTEAALHVDNLAERLLALKGEPVATMREILEKASVREA......NGGESAEAMVASI
A0A132BNU7/1-145 FTLHEKFEELYNETATYIDELAERLLALNGKPIATMKESLETASVKEA......NGNETAEQMVQSV
Q65FU7/1-145 FTLHEKFEELYTEAAEHIDDLAERLLAIQGSPVGTMKECLEMSAIKEA......DGSESAEQMVQNI
D5TXF7/1-147 FTLHEKFEEFYNEAGTYIDELAERILALEGKPLATMKEYLATSSVSEG......TSKESAEEMVQTL
A0A0N0Y8R5/1-151 FELHKKFEEYYNEAAGYIDELAERLLAIGGKPVATMKEYLSLASITEG......TGNETAEQMVNNL
LmDPS_2IY4/1-156 FTLHEKMDDLYSEFGEQMDEVAERLLAIGGSPFSTLKEFLENASVEEAP....YTKPKTMDQLMEDL
SsDps_2XJM/1-165 MIWHPKMDEYMEEIDGYLDEMSERLITLGGAPFSTLKEFSENSQLKEVL....GDYNVTIEEQLARV
SsDps_2UX1/1-165 MIWHPKMDEYMEEIDGYLDEMSERLITLGGAPFSTLKEFSENSQLKEVL....GDYNVTIEEQLARV
EcDps_1L8H/1-167 IAVHEMLDGFRTALICHLATMAERAVQLGGVALGTTQVINSKTPLKSYP.....LDIHNVQDHLKEL
AtDps_1O9R/1-162 IAVHELLDTFRTQLDNHGDTIAERVVQLGGTALGSLQAVSSTTKLKAYP.....TDIYKIHDHLDAL
MsDPS_1UVH/1-183 IGVHEMIDPQVELVRGYADEVAERIATLGKSPKGTPGAIIKDRTWDDYS.....VERDTVQAHLAAL
Rru_A1499/1-187 IVLHRVLIEVATEIETSAHLMASRITALGGDPRDPLQVEADRLFLLFQG.....FGPSDIRQQLLAV
MsDps/1-161 RDLHLQLDELVDFAREGSDTIAERMRALDAVPDGRSDTVAATTTLPEFP.....AFERSTADVVDLI
Rru_A0333/1-158 NTLHLMFEQQYIELSTAVDVIAERIRALGYPAPGSYAQYAKLTVIKEG......SGVPTAEEMIRDL
Cp_symerythrin_3QHC/1-179 PEIGAMFDAIAKEEGMHAQLLTKLYSELEVKDSAETLEAKRLVSTIESQIDAVASDSRGLRRALETA
STAUR_4074/1-157 PEVRAFFLHLAAEEKEHVAEATQMLRMLD............................SAQDGHFAQP
MXAN_3557/1-164 PEVRAFFQHLAAEEKEHVSEAVHMLRMLD............................SGQNDHFAKP
MXAN_4464/1-116 EDAKAILQHVMDEEREHAALFWELIARLD............................PEQAAHAKEA
MXAN_2410/1-109 ASARAELTRLMVEEKARWQRIQELLGGAGG........................RGAAAPTPAREAP
Cthe_1503/1-119 PVAKKVIADIRDEEKAHVGELMTLLRYLD............................PEEAEHFAEG
Tlet_1919/1-116 PLAKKVLIDIANEERVHIGEFTKLLEILT............................KDEAQYMSEG
Mboo_1094/1-124 ELAKAVLTDIANEERVHVGELLRLLSILT............................GNEDEFYKKG
PF1192/1-96 ENVRKILLDVAREEKAHVGEFMALLLNLD............................PEQVTELKGG
PFC_05175short/1-99 ENVRKILLDVAREEKAHVGEFMALLLNLD............................PEQVTELKGG
Hoch_3836/1-131 PGLHDVLIHNKNEEVEHAMMTLEWIRRRS............................PVFDAHMRTY
Rru_A0973/1-140 PELTAILAHNRDEEKEHAAMTLEWLRRND............................AKWAEHLRTY
FRAAL4504/1-136 PELAQVLAHNRDEEKEHAAMTLEWLRRHD............................PVLDTQLRTY
Sce5279/1-127 AELRAVLLHHLEEEVEHAMMNLEWLRRND............................RVFQEKIDIY
WS0286/1-124 PAVRAIMEHNRDEEIEHAVMGLEWLRRHS............................PVWDETMRAL
Amet_4422/1-120 EELKQIMAHNRDEEIEHACMTLEWLRRNM............................DGWDQELKTY
Hore_15960/1-115 PQLKAIVEHNRDEEIEHACMGLEWLRRKY............................PAWHENLKKF
Tpet_0143/1-114 ETVRKILEHNRDEEMEHAAMLLEWLRRNM............................PGWDEALRTY
Tmari_0787/1-114 ETVRKILEHNRDEEMEHAAMLLEWLRRNM............................PGWDEALRTY
Tbd_2465/1-100 PELKAILAHNRDEEKEHAAMVLEWIRRRD............................PCFSEQLKDY
Achr_1378/1-102 PELKAILAHNRDEEKEHASMVLEWIRRKD............................PKFSGELKAY
PG1C_13910/1-99 TELKAILEHNRDEEKEHASMVLEWIQRKD............................PKFSKELYEY
Tgr7_0837/1-101 EELKAILAHNRDEEKEHAAMVLEWIRRRD............................KTFDKELRDY
CYCME_0127/1-95 EELKAILAHNRDEEKEHAAMVLEWIRRKD............................PRMDKELKDY
Q91_0125/1-95 EELKAILAHNRDEEKEHAAMVLEWIRRKD............................PRMDKELKDY
Nmul_A2196/1-96 EELKAILVHNRDEEKEHAAMVLEWIRRKD............................PVFSTELRDY
cce_0034/1-96 KELKAILAHNRDEEKEHAAMLLEWIRRQD............................SVFDEELKDY
Metme_0971/1-95 PELKAILAHNRDEEKEHAAMVLEWIRRKD............................PKFDHELRDY
MEALZ_1148/1-94 KDLKAILAHNRDEEKEHAAMLLEWIRRQD............................PKFDNELRDY
AAY24_01205/1-95 PELKAILAHNRDEEKEHAAMVLEWIRRKD............................PTFDHELKDY
IMCC21906_01077/1-95 PELKRILEHNRDEEKEHAAMTLEWIRRQD............................PVFNDELKDY
Sta7437_0684/1-95 SELKAILSHNRDEEKEHAAMLLEWIRRQD............................PTFDKYLKKY
GU3_08795/1-95 PELKAILKHNRDEEKEHAAMVLEWIRRRD............................PTFDKELKDY
MARPU_03990/1-95 DELRAILAHNRDEEKEHAAMVLEWIRRRD............................PTFDKELKDY
Thivi_3051/1-95 ADLQAILAHNRDEEKEHAAMVLEWIRRKD............................PSFDKELKDY
THII_2135/1-95 AELKAILAHNRDEEKEHAAMVLEWIRRQD............................PAFDKELKDY
NE0167/1-95 KELKAILAHNRDEEKEHAAMLLEWIRRCD............................PAFDKELKDY
Kkor_0109/1-94 NDLRKILRHNRDEEKEHAAMVLEWIRRRD............................PKFDQQLKDY
M911_12845/1-94 AELRSILQHNADEEKEHAAMVLEWIRRRD............................PVFDKELRDW
consensus>70 ....:...ee..:ha...e.l...
H57
E61
E62
E64
H65

```
                                                α3                                        α4
BmBfr_3FVB/1-182                        [helix diagram]
                                                   120                130       140
BmBfr_3FVB/1-182                        LKGEYDA...RASY.KESR.E...........ICDKLGDYVSK.QLFD...............ELL
Rru_A2195/1-159                         LALELAA...RDTL.VEGV.A...........EAEAAQDFVSR.QILD...............EIL
EcBfr_3E1J/1-158                        LALELDG...AKNL.REAI.G...........YADSVHDYVSR.DMMI...............EIL
EcBfr_1BFR/1-158                        LALELDG...AKNL.REAI.G...........YADSVHDYVSR.DMMI...............EIL
S7J5C3/1-158                            LKLELSA...IPDL.RDAI.A...........YAEDIRDYVSR.DLFQ...............DIL
A0A0Q2MUZ5/1-158                        LKLELAA...IPDL.RDAI.A...........YAEDIRDYVSR.DLFQ...............DIL
Q21HY7/1-157                            LKLEMKA...IPDL.RDGI.E...........YAESVRDYISR.DLLQ...............SIL
A0A0F7M3H2/1-159                        LSLELKA...VPDL.KDGI.K...........HCESVADYGSR.ELLE...............EIL
A0A0P1J4Y2/1-159                        LKLEHLA...VPDL.REAM.A...........YCESVQDYVSR.DLFR...............RIL
A0A0C5VE25/1-156                        LKLENMA...VPVL.REAI.A...........YCESVQDYVSR.DLLE...............DIL
A0A0J6N4T8/1-158                        LKLEMDA...LPVL.REAI.A...........YCESAKDYVTR.DLLE...............DIL
A0A0B7DEZ0/1-157                        LRIEKTG...HADL.KAAI.A...........HCETVGDFGSR.ELLE...............DIL
AvBfr_1SOF/1-156                        LKLEQAG...LPDL.KAAI.A...........YCESVGDYASR.ELLE...............DIL
C1DQU9/1-156                            LKLEQAG...LPDL.KAAI.A...........YCESVGDYASR.ELLE...............DIL
A0A0C2S8V2/1-156                        LKLEQSG...IADL.KVAI.A...........YCESVGDYGSR.ELLE...............DIL
PaFtn_3R2M/1-154                        LKLERHV...RAAL.AKGI.A...........LCEQHKDFVSR.DILKA..............QLA
Rru_A3474/1-161                         LDTERTV...VTNL.RRVI.K...........LCEAEKDYVTR.QALLP..............LLN
PmFtn_4ITW/1-168                        LELEQAN...TRSL.LNLA.E...........AASTCHDFAVM.AFLN...............PFH
MsLF_1H96/1-182                         LAMEKNL...NQAL.LDLH.A...........LGSARADPHLC.DFLES..............HYL
Q5R538/1-175                            MALEKKL...NQAL.LDLH.A...........LGSAHTDPHLC.DFLET..............HFL
G3R8I9/1-175                            MALEKKL...NQAL.LDLH.A...........LGSARTDPHLC.DFLET..............HFL
HuLF_2FG4/1-174                         MALEKKL...NQAL.LDLH.A...........LGSARTDPHLC.DFLET..............HFL
E3VX58/1-175                            LALEKNL...NQAL.LDLH.A...........LGSAKTDPHLC.DFLEN..............HFL
Q9JKM8/1-175                            LTLEKSL...NQAL.LDLH.A...........LGSAKTDPHVC.DFLEN..............HFL
L8IKH0/1-175                            LLVEKNL...NQAL.LDLH.G...........LASARGDPHIC.DFLEN..............HFL
B6VQP1/1-175                            IKMEKNL...NEAL.LDLH.A...........LACARADPHLC.DFLES..............HFL
FgMF_3RGD/1-176                         LQLEKTV...NQAL.LDLH.K...........LATDKVDPHLC.DFLES..............EYL
Q6P8C6/1-176                            LQLEKTV...NQAL.LDLH.K...........LASDKTDPHLC.DFLES..............EYL
P85837/1-174                            LQLEKNV...NQSL.LDLH.K...........VCSEHNDPHMC.DFLET..............HYL
P49947/1-176                            LQLEKNV...NQAL.LDLH.K...........IASDKVDPHLC.DFLET..............HYL
HuMF_1R03/1-182                         LLLEKNV...NQSL.LELH.A...........LASDKGDPHLC.DFLET..............YYL
HuHF_2FHA/1-183                         LHLEKNV...NQSL.LELH.K...........LATDKNDPHLC.DFIET..............HYL
MsHF_3WNW/1-212                         LHLEKSV...NQSL.LELH.K...........LATDKNDPHLC.DFIET..............YYL
PgFtn/1-160                             YEHECKV...TEMI.EAVV.R...........AASEAGDMASQ.DFFW...............KYI
EcFtn_1EUM/1-160                        YKHEQLI...TQKI.NELA.H...........AAMTNQDYPTF.NFLQ...............WYV
CjFtn_1KRQ/1-167                        YEHEQFI...TKSI.NTLV.E...........HMLTHKDYSTF.NFLQ...............WYV
HpFtn_3EGM/1-181                        YEHEQHI...SESI.NNIV.D...........HAIKSKDHATF.NFLQ...............WYV
PfFtn_2JD7/1-174                        YEHEKFI...SKSI.YELA.A...........LAEEEKDYSTR.AFLE...............WFI
A0A0C7P5L5/1-169                        IEGENFE...VQEM.YPVYNN...........AAKFQNENEAV.RTTH...............FAL
A0A075WM99/1-170                        IEGESYE...VEEM.YPVFNN...........AAKFQGESEAE.KSTY...............YAL
F8AEI6/1-171                            IEGETYE...VEEM.YPVFKN...........TAELQGEKEAV.RSTH...............YAL
A0A100XZC0/1-171                        IDGETYE...VEEM.YPVFKN...........AAEFQGEKDAI.RTTH...............YAL
Q5JF11/1-171                            IEGETFE...VEEM.YPVYKN...........SAEFQEEKDAV.RTTH...............YAL
Q9V0A0/1-175                            IEGETYE...VEEM.YPVFNK...........TAEFQGEKDAV.RTTH...............FAL
B9KAX2/1-176                            IDGETFE...IEEM.YPVYNT...........VAQFQQEKGAE.RSTK...............FAW
Pf_rubrerythrin_3MPS/1-130              IEGETFE...VEEM.YPVYNK...........AAEFQGEKEAV.RTTH...............YAL
Dv_rubrerythrin_1LKM/1-144              AAGEHHE...YTEM.YPSFAR...........IAREEGYEEIA.RVFA...............SIA
St_sulerythrin_1J30/1-144               IAGETYE...WTQM.YPGFAK...........VAREEGFPEVA.EWFE...............TLA
Bp_rubrerythrin_4DI0/1-144              IAGETHE...YTDM.YPGMAK...........TARDEGFEEIA.NWFE...............TLA
CjDps_3KWO/1-152                        KQDYEYL...LAEF.KKLN.E...........AAEKESDTTTA.AFAQ...............ENI
BbDPS_1N1Q/1-149                        VNDFVDL...VGEL.KVAR.D...........VADEADDEATA.DMLD...............AIE
A0A0M1QIG0/1-146                        VSDFETM...IEEL.KEGM.K...........IADEVGDETTG.DMLL...............GIH
I0BUW7/1-146                            VSDFGIV...IGEL.KEGM.A...........LAEKVGDETTG.DMLL...............AIH
A0A132BNU7/1-145                        YDDFTVI...AEEL.KSGM.D...........LADEVGDETTG.DMLL...............AIH
Q65FU7/1-145                            YNDFLTV...AEEL.KSGM.E...........LADEVNDETTG.DMLL...............AIH
D5TXF7/1-147                            VNDYSAL...IQEL.KEGM.E...........VAGEAGDETSA.DMLL...............AIH
A0A0N0Y8R5/1-151                        ITDFNTL...VDEL.KQGI.D...........IADQAGDESTA.DMLT...............HIR
LmDPS_2IY4/1-156                        VGTLELL...RDEY.QQGI.E...........LTDKEGDNVTN.DMLI...............AFK
SsDps_2XJM/1-165                        VEVFRYL...AALF.QKGF.D...........VSDEEGDSVTN.DIFN...............VAK
SsDps_2UX1/1-165                        VEVFRYL...AALF.QKGF.D...........VSDEEGDSVTN.DIFN...............VAK
EcDps_1L8H/1-167                        ADRYAIV...ANDV.RKAI...............GEAKDDDTA.DILT...............AAS
AtDps_1O9R/1-162                        IERYGEV...ANMI.RKAI.D...........DSDEAGDPTTA.DIFT...............AAS
MsDPS_1UVH/1-183                        DLVYNGV...IEDT.RKSI.............EKLEDLDLVSQ.DLLI...............AHA
Rru_A1499/1-187                         AASLAAF...GHSL.REAI.R...........QAVSIDDAPTA.HLFI...............ELS
MsDps/1-161                             TTRINAT...VDTI.RRVH.............DAVDAEDPSTA.DLLH...............GLI
Rru_A0333/1-158                         ARDQEAV...TRTA.RSIF.P...........VADEAADEPTA.DLLT...............QRM
Cp_symerythrin_3QHC/1-179               LEVETIE...SQKT.YPAFAK...........LAAEQGNMEVA.TAFE...............AIV
STAUR_4074/1-157                        IAPGHFQKVLSAPPA.PPAPASPSPAP.....AGPEQTRAPPVIEPISHLPPQRVVYGVPAP.PST
MXAN_3557/1-164                         FVPGHFQ...AAEAPAPATVH.VPTPDGPAFSVNGRNGRLPSE.PPTS.LPPQRLLYGLPAPPPAV
MXAN_4464/1-116                         VEKYRLI...TSGA.SHEAVE...........AVGKEGAAPS.......PA............DVT
MXAN_2410/1-109                         AEAPRLA...RGSA.DELH.E...........AAP.........FA....................
Cthe_1503/1-119                         EQEVKEM...LEQL..............................NITPK..............PLS
Tlet_1919/1-116                         AKEVEEM........................................................MVE
Mboo_1094/1-124                         TLEVDTL..........................AGQIGAAGAA....................APV
PF1192/1-96                             FEEVQGA...........................................................
PFC_05175short/1-99                     FEEVKEL....TGI.E................A.................................
Hoch_3836/1-131                         LFTERPI....LEL.EEEDTG...........SSSSVAASPT.....................SAP
Rru_A0973/1-140                         LFTEGPI....TAI.EAADTA...........GEGSGGDAAKG...................ATAQ
FRAAL4504/1-136                         LFTSGPV....TEV.EEETMD.....R...GGSGDPDAVTE...................RQAAGP
Sce5279/1-127                           LNSQGPI....TEV.EAAE.............EAAKLGATAKT...................GAMA
WS0286/1-124                            LFKEGEI....ISH.EQAFTG...........KTGEVASALG........................
Amet_4422/1-120                         LFTTGSI....LEA.EMGA.............ETGTETETVV........................
Hore_15960/1-115                        LFSEGDI....ASL.EEEE.............ENQENTETTD........................
Tpet_0143/1-114                         LFTDKPI....TEI.EEET.............SGGSENT...........................
Tmari_0787/1-114                        LFTDKPI....TEI.EEET.............SGGSENT...........................
Tbd_2465/1-100                          LFTDKAI....A...HEAKKD...........S.................................
Achr_1378/1-102                         LFREGPI....N...HEDDAQ...........ASG...............................
PG1C_13910/1-99                         LFTKKTI....A...HRPDDA.............................................
Tgr7_0837/1-101                         LFTDKPI....AGLEHEHKG..............................................
CYCME_0127/1-95                         LFTDKPI....A...HK.................................................
Q91_0125/1-95                           LFTDKPI....A...HK.................................................
Nmul_A2196/1-96                         LFTDKPI....A...HES................................................
cce_0034/1-96                           LFTEKTI....A...HES................................................
Metme_0971/1-95                         LFTDKTI....A...HE.................................................
MEALZ_1148/1-94                         LFTDKPI....A...H..................................................
AAY24_01205/1-95                        LFTEKPI....A...HE.................................................
IMCC21906_01077/1-95                    LFTDKPI....A...HK.................................................
Sta7437_0684/1-95                       LFTSQPI....A...HK.................................................
GU3_08795/1-95                          LFTSGPL....G...HH.................................................
MARPU_03990/1-95                        LFTEKQI....A...HH.................................................
Thivi_3051/1-95                         LFTDKVI....A...HH.................................................
THII_2135/1-95                          LFTNKSI....A...HE.................................................
NE0167/1-95                             LFTNKPI....A...HE.................................................
Kkor_0109/1-94                          LFTEDEI....G...H..................................................
M911_12845/1-94                         LFTDKKL....D...H..................................................
consensus>70                            ...e..............................................................
```

```
                                                                       α5
BmBfr_3FVB/1-182            ℓℓℓℓℓℓℓℓℓℓℓℓℓℓℓℓℓℓℓℓ ℓ..............ℓℓℓℓℓℓ     TT
                              150       160                    170       180

BmBfr_3FVB/1-182            ADEEGHIDFLETQLDLLAKIGG.............ERYGQLNAAPADEAE..............
Rru_A2195/1-159             EDTEEHIDYLETEIGLIDQVGP.............QNYIQSQIDAGES................
EcBfr_3E1J/1-158            RDEEGHIDWLETELDLIQKMGL.............QNYLQAQI..REEG...............
EcBfr_1BFR/1-158            RDEEGHIDWLETELDLIQKMGL.............QNYLQAQI..REEG...............
S7J5C3/1-158                EDEEEHVDWLETQLGLIEMTGI.............ENYLQAQI..VDED...............
A0A0Q2MUZ5/1-158            EDEEEHVDWLETQLGLIDMTGI.............ENYLQAQF..VDED...............
Q21HY7/1-157                DSEEEHVDWLETQLGLIEKIGI.............ENYMQAKM..IKA................
A0A0F7M3H2/1-159            ESEEEHIDWLETQLSLIDKVGL.............QNYQQSMMGGINS................
A0A0P1J4Y2/1-159            ESEEEHIDWLETQLGLIEKVGL.............ENYLQKQMETAED................
A0A0C5VE25/1-156            ESEEEHIDWLETQLDLINKVGI.............ENYLQSHM..DS.................
A0A0J6N4T8/1-158            ESEEEHVDWLETQLSLIESVGL.............QNYLQSQM..HDED...............
A0A0B7DEZ0/1-157            ESEEEHIDWLETQLGLIGKVGL.............ENYLQSQM..GEE................
AvBfr_1SOF/1-156            ESEEDHIDWLETQLDLIDKIGL.............ENYLQSQM..DE.................
C1DQU9/1-156                ESEEDHIDWLETQLDLIDKIGL.............ENYLQSQM..DE.................
A0A0C2S8V2/1-156            ESEEEHIDWLETQLGLIDKVGL.............ENYLQSQM..DD.................
PaFtn_3R2M/1-154            DTEEDHAYWLEQQLGLIARMGL.............ENYLQSQI.....................
Rru_A3474/1-161             DTEEDHLYWLEQQLRLIELTGL.............QNYLQAQMDPAVAA...............
PmFtn_4ITW/1-168            LQQVNEEDKIGSILAKVTDENR.TPGLL..RSLDVVSFLGPCLFRS..................
MsLF_1H96/1-182             DKEVKLIKKMGNHLTNLRRVAGPQPAQTGAPQGSLGEYLFERLTLKHD................
Q5R538/1-175                DEEVKLIKKMGDHLTNLHRLGG........PEAGLGEYLFERLTLKHD................
G3R8I9/1-175                DEEVKLIKKMGDHLTNLHRLGG........PEAGLGEYLFERLTLKHD................
HuLF_2FG4/1-174             DEEVKLIKKMGDHLTNLHRLGG........PEAGLGEYLFERLTLKHD................
E3VX58/1-175                DEEVKLIKKIGDHLTNVRRLAG........PQAGLGEYLFERLTLKHD................
Q9JKM8/1-175                DEEVKLIKKIGDHLTNLRRLDG........PQAGLGEYLFERLTLKHD................
L8IKH0/1-175                DEEVKLIKKRGDHLTNLRRLAG........PQAGLGEYLFERLTLKHD................
B6VQP1/1-175                DEEVKLIKKMGDHLTNLRRLAG........PQAGLGEYLFERLTLKHD................
FgMF_3RGD/1-176             EEQVKDIKRIGDFITNLKRLGL........PENGMGEYLFDKHSVKESS...............
Q6P8C6/1-176                EEQVKAMKELGDYITNLKRLGV........PQNGMGEYLFDKHTLGESS...............
P85837/1-174                DEQVKSIKELGDWVTNLRRLGA........PQNGMAEYLFDKHTLGK.................
P49947/1-176                NEQVEAIKKLGDHITNLTKMDA........VKNKMAEYLFDKHTLGGQS...............
HuMF_1R03/1-182             NEQVKSIKELGDHVHNLVKMGA........PDAGLAEYLFDTHTLGNENKQN............
HuHF_2FHA/1-183             NEQVKAIKELGDHVTNLRKMGA........PESGLAEYLFDKHTLGDSDNES............
MsHF_3WNW/1-212             SEQVKSIKELGDHVTNLRKMGA........PEAGMAEYLFDKHTLGHGDESGDIESAQSDEEVE
PgFtn/1-160                 REQVEEEATAAEIVETIRLSQE............QNLIFIDHQLARR.................
EcFtn_1EUM/1-160            SEQHEEEKLFKSIIDKLSLAGK..........SGEGLYFIDKELST..................
CjFtn_1KRQ/1-167            SEQHEEEALFRGIVDKIKLIGE..........HGNGLYLADQYIKNIALSRKK...........
HpFtn_3EGM/1-181            AEQHEEEVLFKDILDKIELIGN..........ENHGLYLADQYVKGIAKSRKS...........
PfFtn_2JD7/1-174            NEQVEEEASVKKILDKLKFAKD..........SPQILFMLDKELSARAPKLPGLLMQGGE....
A0A0C7P5L5/1-169            EAEKIHEEWYKQAKEATKEEKDIEKGKI..YICDVCGYTVEGEAPDRCPICGAPKSKFSEF...
A0A075WM99/1-170            SAEKIHEELYRKAKELASQEKDMELEKV..FICPVCGFTAVDEAPEFCPVCGTKREMFVEF...
F8AEI6/1-171                EAEKIHAELYAKAKEKAEKGEDIEIKKV..YVCPVCGYTAVDEVPERCPVCGLPKDKFVVFE..
A0A100XZC0/1-171            EAEKIHAELYAKAKEKAESGEDIEVKKV..YICPVCGYTAVDEAPEYCPVCGAPRDKFVIFE..
Q5JF11/1-171                EAEKIHAELYKKAKEKAEKGEDIEIKRV..YICPVCGYTAVDEAPEYCPVCGAPRDKFVVFE..
Q9V0A0/1-175                EAEKIHAELYKKAKESAEQGKDIEIKKV..YICPVCGYTAIDEAPERCPVCGAPRDKFIVFE..
B9KAX2/1-176                EAEKIHAEMYKKAKELVEKGEDYPAEKV..YICPVCGHTVEGEPPEKCPVCGAPRSAYREFSV.
Pf_rubrerythrin_3MPS/1-130  EAEKIHAELYRKAKEKAEKG............................................
Dv_rubrerythrin_1LKM/1-144  VAEEFHEKRFLDFARNIKE.............................................
St_sulerythrin_1J30/1-144   RAEKSHAEKFQNVLKQLKGGT...........................................
Bp_rubrerythrin_4DI0/1-144  KAERSHANRYTKALDGLVD.............................................
CjDps_3KWO/1-152            AKYEKSLWMIGATLQGACKM............................................
BbDPS_1N1Q/1-149            AGLEKHVWMLEAFLE.................................................
A0A0M1QIG0/1-146            QSLEKHVWMLKSFLGR................................................
I0BUW7/1-146                SSLEKHVWMLESFLGK................................................
A0A132BNU7/1-145            QNIEKHNWMLKAYLG.................................................
Q65FU7/1-145                QSIEKHNWMLKAYLG.................................................
D5TXF7/1-147                TTLEQHVWMLSAFLK.................................................
A0A0N0Y8R5/1-151            TSLEKHLWMLNAYMGESQSR............................................
LmDPS_2IY4/1-156            ASIDKHIWMFKAFLGKAPLE............................................
SsDps_2XJM/1-165            ASIEKHIWMLQAELGQAPKL............................................
SsDps_2UX1/1-165            ASIEKHIWMLQAELGQAPKL............................................
EcDps_1L8H/1-167            RDLDKFLWFIESNIE.................................................
AtDps_1O9R/1-162            RDLDKSLWFLEAHVQEKS..............................................
MsDPS_1UVH/1-183            GELEKFQWFVRAHLESAGGQLT.......HEGQSTEKGAADKARRKSA................
Rru_A1499/1-187             RAIDRRFWALDSHISPHERRRE...DLSFDQDQDEGFDPQKPGESQSR................
MsDps/1-161                 DGLEKQAWLIRSENRKV...............................................
Rru_A0333/1-158             QIHEKTAWMLRSMAA.................................................
Cp_symerythrin_3QHC/1-179   KSETKHANWVKRALENLLEVA...........................................
STAUR_4074/1-157            NAAPLTVGSLRRRGGSGNAP............................................
MXAN_3557/1-164             ESHPLTVGSLRRGGGGSGSGR...........................................
MXAN_4464/1-116             PEKRLTVGSLRR....................................................
MXAN_2410/1-109             ..TGLTVGSLRGSR..................................................
Cthe_1503/1-119             GKTESTIGSLIDK...................................................
Tlet_1919/1-116             NNEEPTIGSLKDGEKDGK..............................................
Mboo_1094/1-124             TKEEPTIGSLKNTKEA................................................
PF1192/1-96                 .......................................................NWN......
PFC_05175short/1-99         ................................................................
Hoch_3836/1-131             SHGSLGIGSLRQEGKE..........................................D.....
Rru_A0973/1-140             GDGSLGIGSLKGEAALARPPRL..........................................
FRAAL4504/1-136             GDGSLGIGSLKGVGE.................................................
Sce5279/1-127               PSGALGIGSLKGA...................................................
WS0286/1-124                .SNGLNIGSLKKGK..................................................
Amet_4422/1-120             QEKGLNIGNLKK....................................................
Hore_15960/1-115            ..GSLGLRNTK.....................................................
Tpet_0143/1-114             .GGDLGIRKL......................................................
Tmari_0787/1-114            .GGDLGIRKL......................................................
Tbd_2465/1-100              ................................................................
Achr_1378/1-102             ................................................................
PG1C_13910/1-99             ................................................................
Tgr7_0837/1-101             ................................................................
CYCME_0127/1-95             ................................................................
Q91_0125/1-95               ................................................................
Nmul_A2196/1-96             ................................................................
cce_0034/1-96               ................................................................
Metme_0971/1-95             ................................................................
MEALZ_1148/1-94             ................................................................
AAY24_01205/1-95            ................................................................
IMCC21906_01077/1-95        ................................................................
Sta7437_0684/1-95           ................................................................
GU3_08795/1-95              ................................................................
MARPU_03990/1-95            ................................................................
Thivi_3051/1-95             ................................................................
THII_2135/1-95              ................................................................
NE0167/1-95                 ................................................................
Kkor_0109/1-94              ................................................................
M911_12845/1-94             ................................................................
consensus>70                ................................................................
```

EncFtn C-terminal localization sequence

Amino acid sequences of ferritin family proteins were aligned progressively using EMBL-EBI web services[1,2] Clustal Omega[3], T-Coffee[4] and MAFFT[5]. Protein names were adapted from either UniprotKB[6], KEGG[7] database or common name with PDB entry code[8]. Sequences were sorted in an order corresponding to the clades in phylogenetic tree (Figure 13). The alignment was edited by Espript 3.0 web server[9]. The *Rhodospirillum rubrum* EncFtn (Rru_A0973) sequence was highlighted in yellow. The ferroxidase centre (FOC) of *Pseudo-nitzschia multiseries* ferritin (PmFtn_4ITW) (highlighting in blue) consists of Fe_A site (E16, E49, E52) and Fe_B site (E49, E95, E131, Q128) which were labelled with solid red triangles[10]. Another iron binding site in PmFtn_4ITW (the gateway site or Fe_C site[11]) consisted of E48, E45 and E131 which are marked with solid blue circles[10]. The FOC of *R. rubrum* EncFtn was labelled with empty red triangles as E32, E62, H65 and Y39; and the iron entry site was marked with empty blue circles including E31 and E34.The putative iron exit site was marked with empty blue squares including H57, E61 and E64. The C-terminal localization sequences common to the encapsulin associated ferritins were highlighted within the red rectangle.

References

1. Li, W. *et al.* The EMBL-EBI bioinformatics web and programmatic tools framework. *Nucleic Acids Res.* **43,** W580–4 (2015).
2. McWilliam, H. *et al.* Analysis Tool Web Services from the EMBL-EBI. *Nucleic Acids Res.* **41,** (2013).
3. Sievers, F. *et al.* Fast, scalable generation of high-quality protein multiple sequence alignments using Clustal Omega. *Mol. Syst. Biol.* **7,** 539 (2011).
4. Notredame, C., Higgins, D. G. & Heringa, J. T-Coffee: A novel method for fast and accurate multiple sequence alignment. *J. Mol. Biol.* **302,** 205–17 (2000).
5. Katoh, K. & Standley, D. M. MAFFT multiple sequence alignment software version 7: Improvements in performance and usability. *Mol. Biol. Evol.* **30,** 772–780 (2013).
6. The UniProt Consortium. UniProt: a hub for protein information. *Nucleic Acids Res.* **43,** D204–12 (2014).
7. Ogata, H. *et al.* KEGG: Kyoto encyclopedia of genes and genomes. *Nucleic Acids Research* **27,** 29–34 (1999).
8. Berman, H. M. *et al.* The Protein Data Bank. *Nucleic Acids Res.* **28,** 235–242 (2000).
9. Robert, X. & Gouet, P. Deciphering key features in protein structures with the new ENDscript server. *Nucleic Acids Res.* **42,** (2014).
10. Pfaffen, S., Abdulqadir, R., Le Brun, N. E. & Murphy, M. E. P. Mechanism of ferrous iron binding and oxidation by ferritin from a pennate diatom. *J. Biol. Chem.* **288,** 14917–25 (2013).
11. Ebrahimi, K. H. Unity in the Biochemistry of the Iron-Storage Proteins Ferritin and Bacterioferritin. *Chem. Rev.* (2014).
