# Supplementary material for: Structural characterization of encapsulated ferritin provides insight into iron storage in bacterial nanocompartments
Source: eLife. 2016 Aug 16;5:e18972. doi: 10.7554/eLife.18972 (PMC5012862; doi:10.7554/eLife.18972)
Supplement: Supplementary file 2. — Primers used to generate the original constructs used in this study are listed 5’ to 3’, from left to right. Introduced restriction sites are shown underlined; regions complimentary to genomic DNA shown in bold. DOI: http://dx.doi.org/10.7554/eLife.18972.036 [file elife-18972-supp2.docx]

**Supplementary File 2. Primers used in this study.**

| **Protein acronym** | **Construct** | **Forward primer** | **Reverse primer** | **Restriction sites** | **Tag** |
| --- | --- | --- | --- | --- | --- |
| EncFtn_sH_ | pET-28a-Rru_A0973_1-96His6_ | GGCCCATG**GCGCAGTCGAGCAATAGCACC** | AAGGAATTCGC**CGCGGTGATCGGCCCCTC** | EcoRI  NcoI | C-His_6_ |
| EncFtn | pACYCDuet-1-Rru_A0973 | GGCCCATG**GCGCAGTCGAGCAATAGCACC** | GGCGAATTC**TTACAACCGGGGTGGACGGG** | EcoRI  NcoI | None |
| Encapsulin | pACYCDuet-1-Rru_A0974 | GTGTATTCCAT**ATGAACGATCTGATGCGTGACTTG** | TATGGTACC**TTATGCCTTGGTGGCGGC** | KpnI  NdeI | None |
| EncFtn-Enc | pACYCDuet-1-Rru_A0973-Rru_A0974 | GGCCCATG**GCGCAGTCGAGCAATAGCACC** | GGCGAATTC**TTACAACCGGGGTGGACGGG** | EcoRI  NcoI | None |
|  |  | GTGTATTCCAT**ATGAACGATCTGATGCGTGACTTG** | TATGGTACC**TTATGCCTTGGTGGCGGC** | KpnI  NdeI | None |

All primers are listed 5’ to 3’, from left to right. Introduced restriction sites are shown underlined; regions complimentary to genomic DNA shown in bold.
